# Supplementary material for: Microbiota regulates the TET1-mediated DNA hydroxymethylation program in innate lymphoid cell differentiation
Source: Nat Commun. 2024 Jun 5;15:4792. doi: 10.1038/s41467-024-48794-0 (PMC11153590; doi:10.1038/s41467-024-48794-0)
Supplement: Supplementary file 1 — Supplementary Information [file 41467_2024_48794_MOESM1_ESM.pdf]

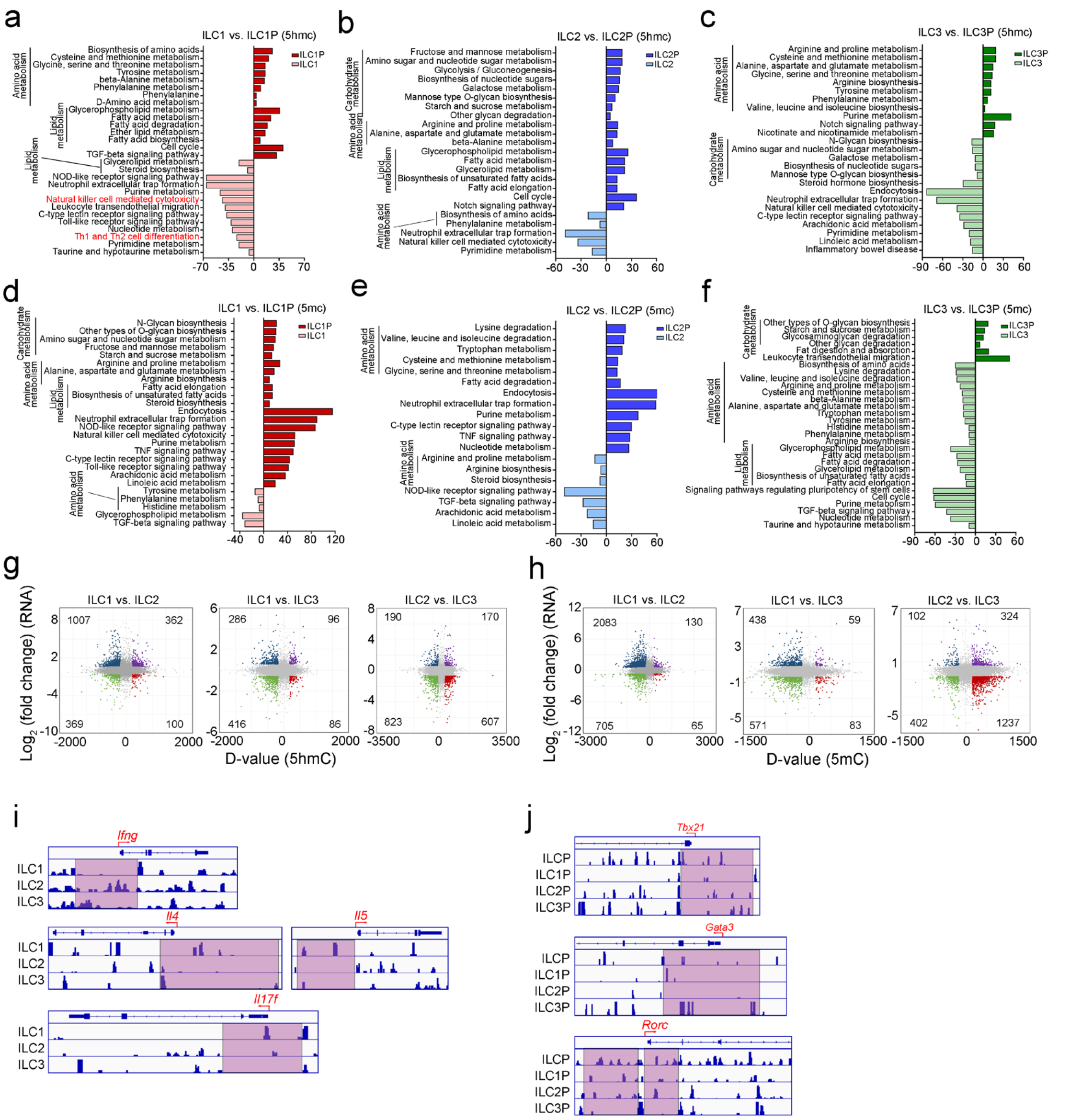

Supplementary Fig. 1. Analysis of methylation and hydroxymethylation of promoters in different ILC subsets.

(a-c) KEGG enrichment of genes with hyper-hydroxymethylated promoters in each ILC subset comparing with their respective precursors. KEGG database was utilized to annotate the genes with hyper-hydroxymethylated promoters in ILC1s, ILC2s and ILC3s as well as their respective precursors. The pathways related to carbohydrate metabolism, lipid metabolism and amino acid metabolism were identified. (d-f) KEGG enrichment of genes with hypermethylated promoters in each ILC subset and their respective precursors. KEGG database was utilized to annotate the genes with hypermethylated promoters in ILC1s, ILC2s and ILC3s as well as their respective precursors. The pathways related to carbohydrate metabolism, lipid metabolism and amino acid metabolism were identified. (g) Analysis of differentially hydroxymethylated promoters (DHMPs) and expression of their respective genes between each pair of ILC subsets. The D-value of DHMPs and $\log_2$ (fold change) of expression level of respective genes between each pair of ILC subsets are shown. The transcriptome data were from the GEO database (GSE37448). (h) Analysis of differentially methylated promoters (DMPs) and expression of their respective genes between each pair of

ILC subsets. The D-value of DMPs and log_2 (fold change) of expression level of respective genes between each pair of ILC subsets are shown. (i) The distribution of 5mC in cytokine genes in ILC1s, ILC2s and ILC3s. The methylated peaks across genes including *Ifng*, *Il4*, *Il5* and *Il17f* of each ILC subset were visualized by IGV. (j) The distribution of 5mC across genes of lineage-specific transcription factors in ILCPs, ILC1Ps, ILC2Ps and ILC3Ps. The methylated peaks across genes including *Tbx21*, *Gata3* and *Rorc* of each cell subset were visualized by IGV. Source data are provided as a Source Data file.

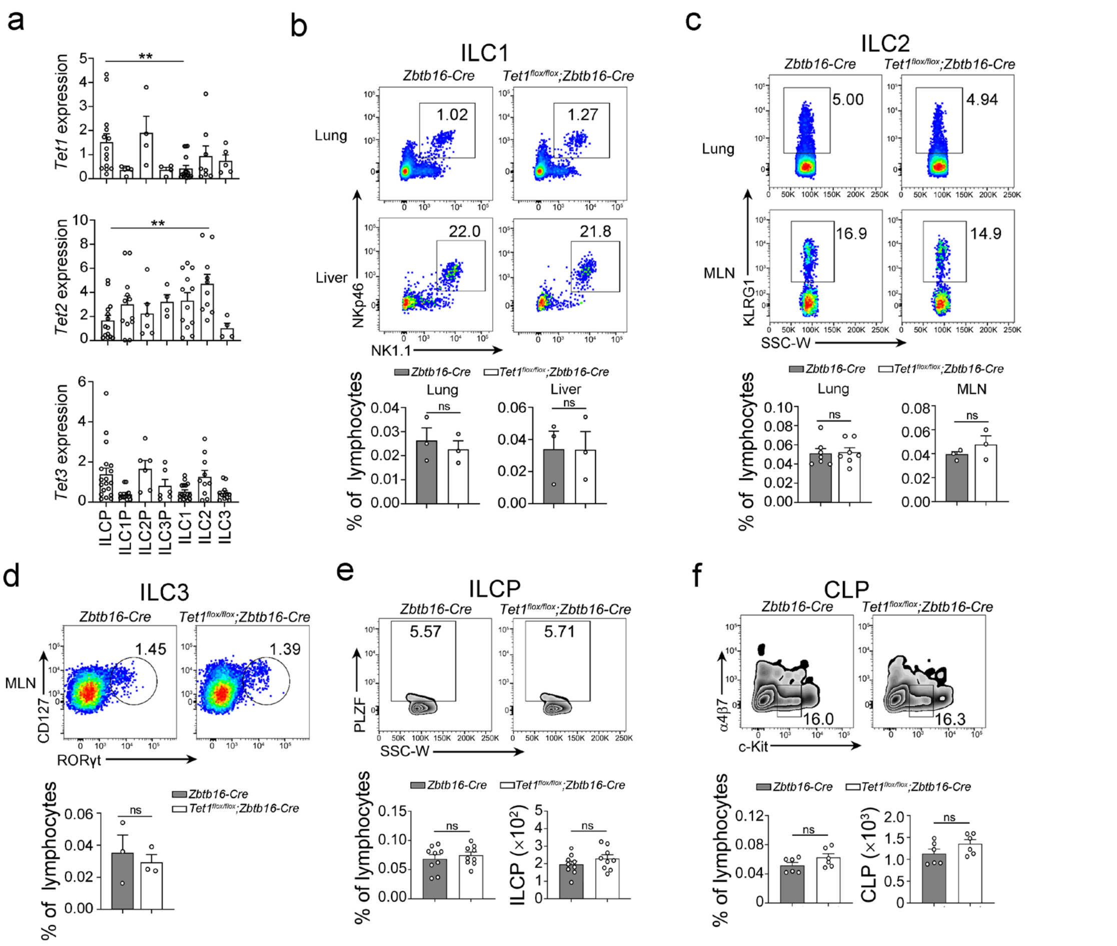

Supplementary Fig. 2. The effect of TET1 on the differentiation of ILC subsets in other tissues.

(a) Gene expression of *Tet1*, *Tet2* and *Tet3* in ILC subsets. ILC1s, ILC2s and ILC3s from the small intestine and ILC1 precursors (ILC1Ps), ILC2 precursors (ILC2Ps), ILC3 precursors (ILC3Ps) and common ILC precursors (ILCPs) from the bone marrow (BM) of mice were collected for qPCR assays. The relative expression of the indicated genes is shown as the mean ± SEM. **, $p < 0.01$ by one-way ANOVA ($p = 0.0048, 0.0015$). (*Tet1* expression: n = 14 for ILCPs, n = 5 for ILC1Ps, n = 4 for ILC2Ps, n = 4 for ILC3Ps, n = 16 for ILC1s, n = 8 for ILC2s, n = 5 for ILC3s; *Tet2* expression: n = 14 for ILCPs, n = 12 for ILC1Ps, n = 6 for ILC2Ps, n = 4 for ILC3Ps, n = 12 for ILC1s, n = 10 for ILC2s, n = 4 for ILC3s; *Tet3* expression: n = 19 for ILCPs, n = 12 for ILC1Ps, n = 6 for ILC2Ps, n = 6 for ILC3Ps, n = 16 for ILC1s, n = 10 for ILC2s, n = 12 for ILC3s). (b) Flow cytometry analysis of ILC1s in the lung and liver from *Zbtb16-Cre* and *Tet1*$^{flox/flox}$*;Zbtb16-Cre* mice. The percentage of ILC1s is shown as mean ± SEM. ns, not significant by two-sided unpaired Student's *t* test ($p = 0.59, 0.52$). n = 3 for each group. (c) Flow cytometry analysis of ILC2s in the lung (n = 7) and mesenteric lymph node (MLN) (n = 3) from *Zbtb16-Cre* and *Tet1*$^{flox/flox}$*;Zbtb16-Cre* mice. The percentage of ILC2s is shown as the mean ± SEM. ns, not significant by two-sided unpaired Student's *t* test ($p = 0.86, 0.35$). (d) Flow cytometry analysis of ILC3s in MLN from *Zbtb16-Cre* and *Tet1*$^{flox/flox}$*;Zbtb16-Cre* mice. The percentage of ILC3s is shown as the mean ± SEM. ns, not significant by two-sided unpaired Student's *t* test ($p = 0.66$). n=3 for each group. (e) Flow cytometry analysis of ILCPs in bone marrow (BM) from *Zbtb16-Cre* and *Tet1*$^{flox/flox}$*;Zbtb16-Cre* mice. The percentage and cell number of ILCPs is shown as the mean

± SEM. ns, not significant by two-sided unpaired Student's *t* test (p = 0.50, 0.25). n = 9 for each group. (f) Flow cytometry analysis of common lymphoid progenitors (CLPs) (CLP = $Lin^{-}CD127^{+}Sca\text{-}1^{+}c\text{-}Kit^{low}\alpha4\beta7^{-}$, Lin = CD3, CD19) in bone marrow (BM) from *Zbtb16-Cre* and *Tet1*$^{flox/flox}$*;Zbtb16-Cre* mice. The percentage and cell number of CLPs is shown as the mean ± SEM. ns, not significant by two-sided unpaired Student's *t* test (p = 0.13, 0.14). n = 6 for each group. All data are representative of at least three independent experiments. Source data are provided as a Source Data file.

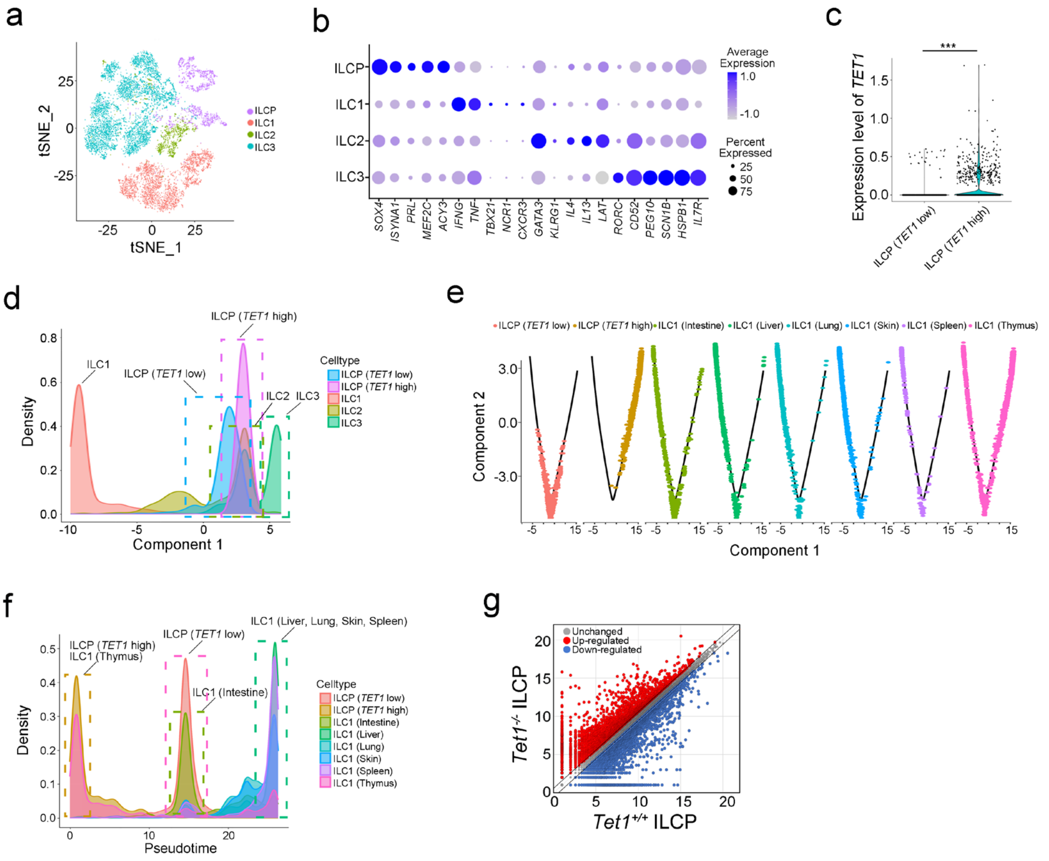

Supplementary Fig. 3. Human ILCPs with low expression of TET1 are prone to differentiate to ILC1s.

(a) Single-cell transcriptome data of human ILC subsets were from the GEO database (GSE163587). ILC subsets were determined and shown by t-distributed stochastic neighbor embedding (t-SNE). Four ILC clusters (including ILC1s, ILC2s, ILC3s and ILCPs) were identified. (b) Dot plot for expression of featured genes in identified ILC subsets. Color represents the average level of gene expression in cells, while size represents the proportion of cells that express these genes. (c) Violin plots show the expression level of *TET1* in ILCPs subgroups. The ILCPs were divided into ILCP (*TET1* low) and ILCP (*TET1* high) subgroups according to *TET1* expression. The expression of *TET1* is shown as mean ± SEM. ***, $p <$ 0.001 by two-sided unpaired Student's *t* test. n = 810 for ILCPs (*TET1* low) and 1006 for ILCPs (*TET1* high). (d) Quantification of pseudotime plot comparison by different ILC subsets. The component 1 of differentiation trajectory of ILCPs (*TET1* low or *TET1* high), ILC1s, ILC2s and ILC3s were analyzed by Monocle2. (e-f) Trajectory analysis of differentiation of ILCPs to ILC1s in different tissues. Trajectory analysis (e) and density plot (f) were used to analyze the differentiation of ILCPs (*TET1* low or *TET1* high) to ILC1s in intestine, liver, lung, skin, spleen and thymus. (g) Transcriptome analysis of bone marrow ILCPs from *Zbtb16-Cre* mice and *Tet1*$^{flox/flox}$*;Zbtb16-Cre* mice. The M-versus-A (MA) plot displays the up-(red) and down-(blue) regulated genes in *Tet1*$^{-/-}$ILCPs compared with *Tet1*$^{+/+}$ ILCPs. The differentially expressed genes (DEGs) were determined by EdgeR analysis. Source data are provided as a Source Data file.

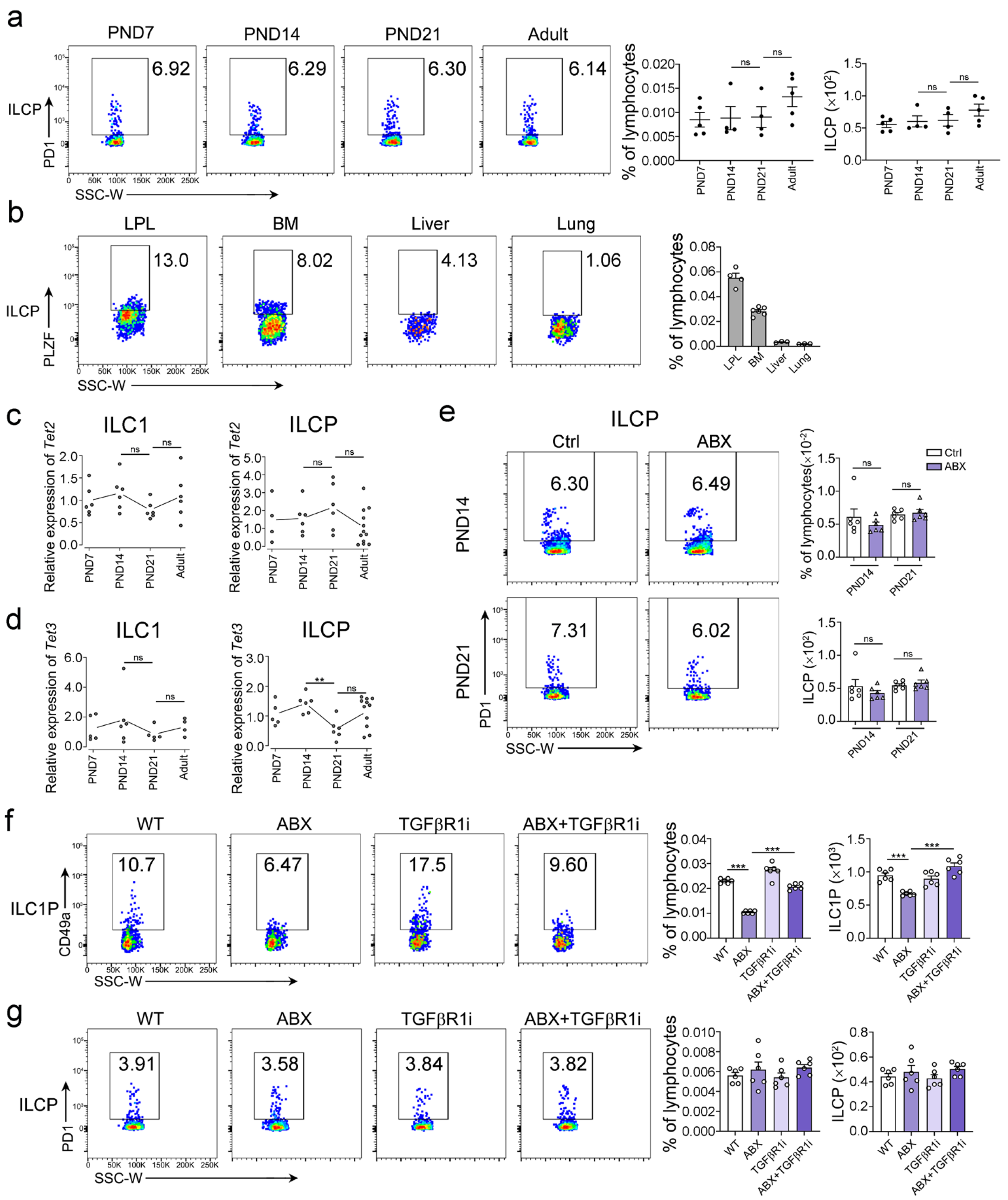

Supplementary Fig. 4. Gut commensal bacteria promote ILC1s differentiation during the postnatal stage.

(a) Bone marrow (BM)-derived ILCPs from C57BL/6 mice at the indicated postnatal stages (from postnatal Day 7 (PND7) to adulthood) were analyzed by flow cytometry. The percentage and cell number of ILCPs were analyzed by flow cytometry and shown as the mean ± SEM. ns, not significant by one-way ANOVA (p = 0.99, 0.48, 0.99, 0.52). (n = 5 for PND7, n = 4 for PND14, n = 4 for PND21, n = 5 for Adult). (b) Flow cytometry analysis of ILCPs in the different tissues of PLZF-GFP reporter mice. The percentages of ILCPs in lamina propria lymphocytes (LPL), BM, liver and lung were calculated and shown as mean ± SEM. (n = 4 for LPL, n = 6 for BM, n = 3 for Liver , n = 3 for Lung). (c-d) Gene expression of *Tet2* and *Tet3* in ILC1s and ILCPs of C57BL/6 mice at the indicated postnatal stages were detected by qPCR. ILC1s and ILCPs were isolated from the gut and BM for qPCR assay, respectively. The gene expression of *Tet2* (c) and *Tet3* (d) in ILC1s and ILCPs is shown as the mean ± SEM. **, p < 0.01; ns, not

significant by one-way ANOVA (p = 0.33, 0.52, 0.75, 0.20 for c, p = 0.51, 0.91, 0.0096, 0,054 for d). (*Tet2* expression in ILC1s: n = 6 for each group; *Tet2* expression in ILCPs: n = 4 for PND7, n = 6 for PND14, n = 6 for PND21, n = 12 for Adult; *Tet3* expression in ILC1s: n = 5 for PND7, n = 6 for PND14, n = 5 for PND21, n = 4 for Adult; *Tet3* expression in ILCPs: n = 5 for PND7, n = 6 for PND14, n = 6 for PND21, n = 11 for Adult). (e) The BM-derived ILCPs from C57BL/6 mice after ABX treatment were analyzed by flow cytometry. C57BL/6 mice were oral gavaged with 100 μL of PBS (Ctrl) or antibiotic mixture (ABX, 0.05 g/mL ampicillin, vancomycin, metronidazole, neomycin and streptomycin sulfate) at PND7 for 14 days. The percentage and cell number of ILCPs are shown as the mean ± SEM. ns, not significant by two-sided unpaired Student's *t* test (p = 0.37, 0.66, 0.37, 0.47). n = 6 for each group. (f-g) Intestinal microbiota promotes the expansion of ILC1Ps via TGF-β signaling. Mice were orally gavaged with ABX and intraperitoneally injected with 200 μL of 1 mg/mL TGF-βR1 inhibitor (TGFβR1i) at PND7 twice a week for two weeks. The percentage and cell number of ILC1Ps (f) and ILCPs (g) were analyzed by flow cytometry and shown as the mean ± SEM. ***, $p < 0.001$ by one-way ANOVA. n = 6 for each group. All data represented at least three independent experiments. Source data are provided as a Source Data file.

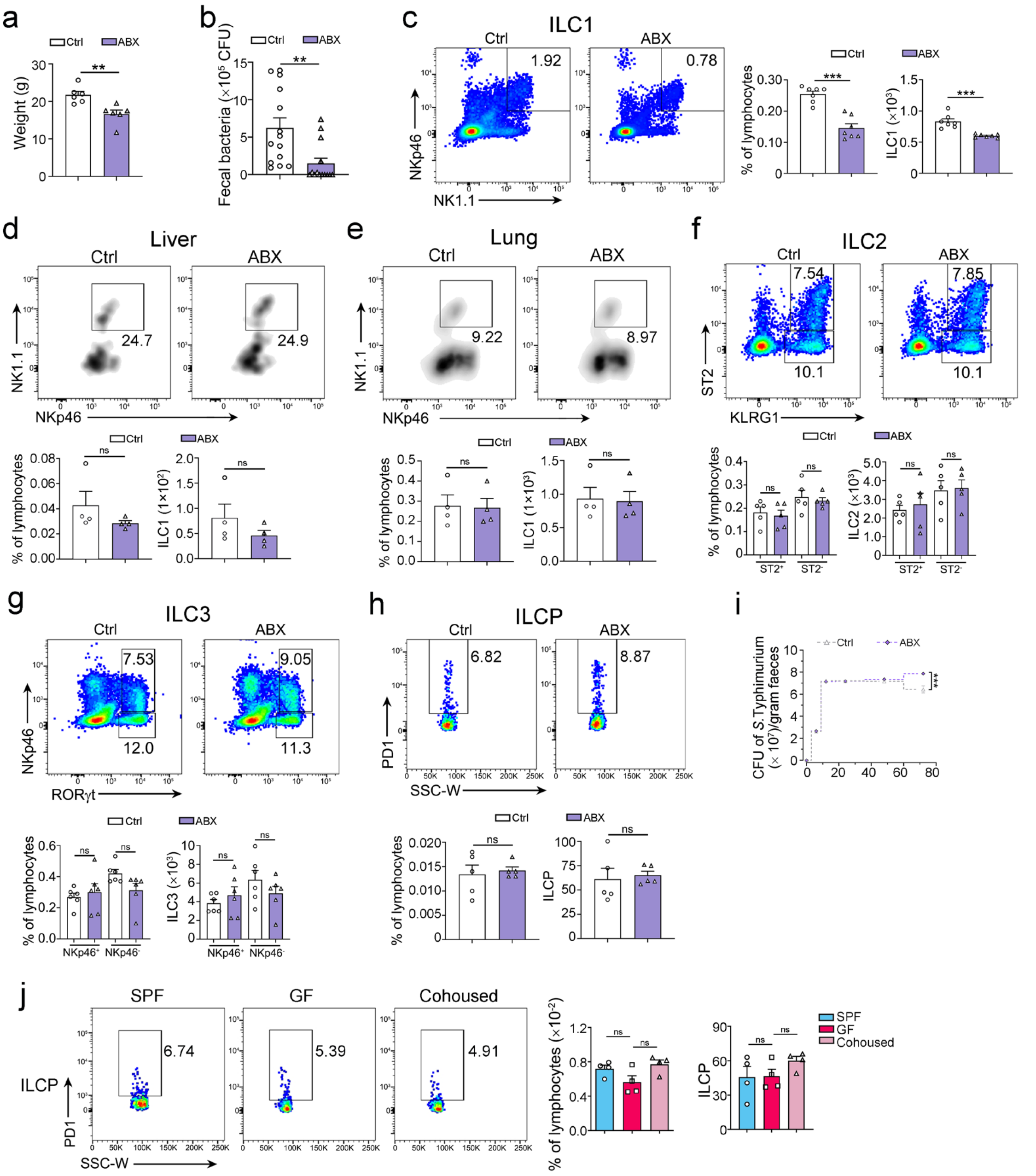

Supplementary Fig. 5. Gut commensal bacteria promote ILC1 differentiation.

(a-b) Changes in body weight and fecal microbiota were observed in adult mice after antibiotic treatment. The WT C57BL/6 mice were treated with PBS (Ctrl) or antibiotic mixture (ABX) from postnatal day 7 to adulthood. The body weight (a) (n = 6 for each group) and fecal microbiota (b) (n = 14 for each group) of mice were analyzed at 8 weeks of age and are shown as the mean ± SEM. **, $p < 0.01$ by two-sided unpaired Student's *t* test ($p = 0.0045$ for a; $p = 0.0051$ for b). (c) Flow cytometry analysis of ILC1s in small intestine from adult mice after ABX treatment as described above. The percentage and cell number of ILC1s are shown as the mean ± SEM. ***, $p < 0.001$ by two-sided unpaired Student's *t* test. n = 7 for each group. (d-e) Flow cytometry analysis of ILC1s in liver and lung from WT adult mice after ABX treatment. The C57BL/6 mice were treated with PBS (Ctrl) or antibiotic mixture (ABX) from

postnatal day 7 to adulthood. The percentage and cell number of ILC1s in liver (d) and lung (e) are shown as the mean ± SEM. ns, not significant by two-sided unpaired Student's *t* test (p = 0.25, 0.28 for d, p = 0.89, 0.87 for e). n = 4 for each group. (f-g) Flow cytometry analysis of ILC2s and ILC3s in small intestine from WT adult mice after ABX treatment as described above. The percentage and cell number of ILC2s (f) (n = 5 for each group) and ILC3s (g) (n = 6 for each group) are shown as the mean ± SEM. ns, not significant by two-sided unpaired Student's *t* test (p = 0.68, 0.62, 0.66, 0.85 for f, p = 0.61, 0.06, 0.41, 0.27 for g). (h) Flow cytometry analysis of ILCPs in BM from adult mice after ABX treatment. The percentage and cell number of ILCPs are shown as the mean ± SEM. ns, not significant by two-sided unpaired Student's *t* test (p = 0.67, 0.76). n = 5 for each group. (i) ABX treatment made mice more susceptible to *S.* Typhimurium infection. The feces were collected from mice with or without ABX treatment after 0 hour (h) (n = 6), 6 hours (h) (n = 8), 12 h (n = 4), 24 h (n = 4), 48 h (n = 8) and 72 h (n = 8) of streptomycin-resistant *Salmonella enterica* serovar Typhimurium (*S.* Typhimurium) infection (1×10^7 CFU per mouse). *S.* Typhimurium in feces were cultured on the plates with media containing 2 mg/mL streptomycin and counted after 72 hours. The CFU of fecal *S.* Typhimurium are shown as the mean ± SEM. ***, $p < 0.001$ by two-sided unpaired Student's *t* test. (j) Gut microbiota did not affect ILCP population in BM. WT mice under SPF, germ-free (GF) or germ-free conditions followed by cohousing with SPF mice for two weeks (Cohoused) were sacrificed for flow cytometry analysis of ILCPs (ILCPs = $Lin^-c\text{-}Kit^+CD127^+\alpha4\beta7^+PD1^+$, Lin = CD3, CD19, CD11b, Gr1, Ter119, CD45R). The percentage and cell number of ILCPs were analyzed by flow cytometry and shown as the mean ± SEM. ns, not significant by two-sided unpaired Student's *t* test (p = 0.11, 0.06, 0.95, 0.11). n = 4 for each group. All data represented at least three independent experiments. Source data are provided as a Source Data file.

Supplementary Table 1: The primers used for real-time RT-PCR and ChIP-qPCR.

Gene	Sequence (5'-3')
Tgfbr1-chip1-F	AAGTGATTGGACGCCCTCTT
Tgfbr1-chip1-R	CCCCACCCCTAAAGCCTTG
Tgfbr1-chip2-F	CTCTTAGCCCCCACGGTTTG
Tgfbr1-chip2-R	GGAATCAGCCGGCCTCTAGT
Tgfbr1-chip3-F	GTACCTATGACTGCTCCCGC
Tgfbr1-chip3-R	TGTGTCTCGGCACCGTAAAA
Tgfbr1-chip4-F	CGGAAAAATGCTGTCGGTGC
Tgfbr1-chip4-R	TTTACTGTGAGACGCTCCACC
Tgfbr1-chip5-F	CGCTGTGGCTGGAGTAAAGG
Tgfbr1-chip5-R	GGGTTTCTGCCTCTAACCACG
Tgfbr1-chip6-F	TTCCTGAGGAGAAGCTGCG
Tgfbr1-chip6-R	GAGGAGCTGCGGACGAC
*Tgfbr1*_F	TCTGCATTGCACTTATGCTGA
*Tgfbr1*_R	AAAGGGCGATCTAGTGATGGA
*Tgfbr2*_F	CCGCTGCATATCGTCCTGTG
*Tgfbr2*_R	AGTGGATGGATGGTCCTATTACA
*Tgfbr3*_F	GGTGTGAACTGTCACCGATCA
*Tgfbr3*_R	GTTTAGGATGTGAACCTCCCTTG
16s-F	GTGSTGCAYGGYTGTCGTCA
16s-R	ACGTCRTCCMCACCTTCCTC
Tet1-F	GAAGGAACAGGAAGCTGCAC
Tet1-R	CTGGCCAAACCTAGTCTCCA
Tet2-F	GATCCAGGAGGAGCAGTGAG
Tet2-R	TGGGAGAAGGTGGTGCTATC
Tet3-F	CCGGATTGAGAAGGTCATCTAC
Tet3-R	AAGATAACAATCACGGCGTTCT
Gpbar1-F	CCTGGCAAGCCTCATCGTC
Gpbar1-R	AGCAGCCCGGCTAGTAGTAG
Nr1h4-F	GCTTGATGTGCTACAAAAGCTG
Nr1h4-R	CGTGGTGATGGTTGAATGTCC
Nr1i1-F	ACCCTGGTGACTTTGACCG

Nr1i1-R	GGCAATCTCCATTGAAGGGG
Nr1i2-F	GATGGAGGTCTTCAAATCTGCC
Nr1i2-R	GGCCCTTCTGAAAAACCCCT
Nr1i3-F	CCCTGACAGACCCGGAGTTA
Nr1i3-R	GCCGAGACTGTTGTTCCATAAT
Gapdh-F	AGGTCGGTGTGAACGGATTTG
Gapdh-R	TGTAGACCATGTAGTTGAGGTCA
Tgfbr1-p-F	CACAGTTCCACTCCAGGTGTC
Tgfbr1-p-R	GACACACCTAAAATCTCCAGTG
Tgfbr1-gb-F	GACTTTTCTTCAACCAGGGG
Tgfbr1-gb-R	CCTTCTGGTTACTGGACTCT
