# Supplementary material for: Microbiota regulates the TET1-mediated DNA hydroxymethylation program in innate lymphoid cell differentiation
Source: Nat Commun. 2024 Jun 5;15:4792. doi: 10.1038/s41467-024-48794-0 (PMC11153590; doi:10.1038/s41467-024-48794-0)
Supplement: Supplementary file 3 — Reporting Summary [file 41467_2024_48794_MOESM3_ESM.pdf]

nature portfolio

Corresponding author(s): Shuo Wang
Last updated by author(s): Feb-21-2024

Reporting Summary

Nature Portfolio wishes to improve the reproducibility of the work that we publish. This form provides structure for consistency and transparency in reporting. For further information on Nature Portfolio policies, see our Editorial Policies and the Editorial Policy Checklist.

Statistics

For all statistical analyses, confirm that the following items are present in the figure legend, table legend, main text, or Methods section.

n/a	Confirmed	
☐	☒	The exact sample size (*n*) for each experimental group/condition, given as a discrete number and unit of measurement
☐	☒	A statement on whether measurements were taken from distinct samples or whether the same sample was measured repeatedly
☐	☒	The statistical test(s) used AND whether they are one- or two-sided<br>*Only common tests should be described solely by name; describe more complex techniques in the Methods section.*
☒	☐	A description of all covariates tested
☐	☒	A description of any assumptions or corrections, such as tests of normality and adjustment for multiple comparisons
☐	☒	A full description of the statistical parameters including central tendency (e.g. means) or other basic estimates (e.g. regression coefficient) AND variation (e.g. standard deviation) or associated estimates of uncertainty (e.g. confidence intervals)
☐	☒	For null hypothesis testing, the test statistic (e.g. *F*, *t*, *r*) with confidence intervals, effect sizes, degrees of freedom and *P* value noted<br>*Give P values as exact values whenever suitable.*
☒	☐	For Bayesian analysis, information on the choice of priors and Markov chain Monte Carlo settings
☒	☐	For hierarchical and complex designs, identification of the appropriate level for tests and full reporting of outcomes
☒	☐	Estimates of effect sizes (e.g. Cohen's *d*, Pearson's *r*), indicating how they were calculated

Our web collection on statistics for biologists contains articles on many of the points above.

Software and code

Policy information about availability of computer code

Data collection	For MeDIP-seq and hMeDIP-seq, we aligned reads to the mouse mm10 genome by using Bowtie2 software (version 2.4.2) to generate the bam file; For RAN-seq, Fastqc (Version 0.11.9) was used to evaluate raw reads for quality control, followed by trimming sequence with Trimmomatic (Version 0.39). we aligned reads to the mouse GRCm39 genome by using Hisat2 software (version 2.1.0) and samtools (version 1.9) to generate the bam file;
Data analysis	For MeDIP-seq and hMeDIP-seq, peaks were called by MACS2 (Version 2.2.7) and visualized by IGV (Version 2.9.4), ChIPseeker and ClusterProfiler packages of R (version 4.1.2). Annotation of peaks was performed with Homer; For RAN-seq, StringTie (Version 2.2.1) was used to assemble and quantitate transcripts. Differentially expressed genes were analyzed and plotted using EdgeR (Version 3.14) and ggplot2 packages of R, respectively. Gene Set Enrichment Analysis was performed with GSEA (Version 4.2.2); For 16S rRNA sequencing, Cutadapt (Version 1.9.1) and UCHIME algorithm were used to obtain clean reads. The clean reads were assigned to the same OTUs with ≥ 97% similarity by using Uparse software (version 7.0.1001) . The α-diversity indexes (including ACE, Chao1, Shannon and Observed species) were calculated using MOTHUR program. QIIME 2 (version 2021.4) was used to calculate weighted-UniFrad distances of PCoA and Shannon index. Functional prediction of the microbiota was performed by using the Bugbase package of R; For metabolomics, Analyst TF (Version 1.7) software was used to screen molecule and ion >100 and collect secondary mass spectrometric data. The Progenesis QI (Version 2.3) software was used for raw peak exacting, data baseline filtering and calibration, peak alignment, peak identification, and peak area integration; For flow cytometry data analysis, we respectively used FlowJo (version 10.0) and GraphPad Prism (version 9.0.0) to gated targeted cell populations and performe statistic analysis.

For manuscripts utilizing custom algorithms or software that are central to the research but not yet described in published literature, software must be made available to editors and reviewers. We strongly encourage code deposition in a community repository (e.g. GitHub). See the Nature Portfolio guidelines for submitting code & software for further information.

Data

Policy information about availability of data

All manuscripts must include a data availability statement. This statement should provide the following information, where applicable:

- Accession codes, unique identifiers, or web links for publicly available datasets
- A description of any restrictions on data availability
- For clinical datasets or third party data, please ensure that the statement adheres to our policy

Data are deposited to China National Microbiology Data Center (NMDC) (https://nmdc.cn/resource/) (accession numbers: PRJCA020429 and PRJCA025083). Source data are provided with this paper. M. musculus reference genome (mm10) was downloaded from UCSS Genome Browser database (https://genome.ucsc.edu).

Research involving human participants, their data, or biological material

Policy information about studies with human participants or human data. See also policy information about sex, gender (identity/presentation), and sexual orientation and race, ethnicity and racism.

Reporting on sex and gender	N/A
Reporting on race, ethnicity, or other socially relevant groupings	N/A
Population characteristics	N/A
Recruitment	N/A
Ethics oversight	N/A

Note that full information on the approval of the study protocol must also be provided in the manuscript.

Field-specific reporting

Please select the one below that is the best fit for your research. If you are not sure, read the appropriate sections before making your selection.

- [x] Life sciences
- [ ] Behavioural & social sciences
- [ ] Ecological, evolutionary & environmental sciences

For a reference copy of the document with all sections, see nature.com/documents/nr-reporting-summary-flat.pdf

Life sciences study design

All studies must disclose on these points even when the disclosure is negative.

Sample size	Sample sizes for mouse studies were selected so as to power non-parametric statistical analyses
Data exclusions	No data were excluded from the analyses.
Replication	Data were either pooled from at least three replicate experiments; or representative data are shown. The number of biological replicates were provided in the figure legends.
Randomization	Allocation of mice to treatment groups was randomized.
Blinding	No blinding was performed. The samples were prepared, treated and analysed by the same standard procedure. The investigators did not expect the experimental results and most of the test data was automatically generated by the instrument

Reporting for specific materials, systems and methods

We require information from authors about some types of materials, experimental systems and methods used in many studies. Here, indicate whether each material, system or method listed is relevant to your study. If you are not sure if a list item applies to your research, read the appropriate section before selecting a response.

Materials & experimental systems

n/a	Involved in the study
☐	☒ Antibodies
☐	☒ Eukaryotic cell lines
☒	☐ Palaeontology and archaeology
☐	☒ Animals and other organisms
☒	☐ Clinical data
☒	☐ Dual use research of concern
☒	☐ Plants

Methods

n/a	Involved in the study
☒	☐ ChIP-seq
☐	☒ Flow cytometry
☒	☐ MRI-based neuroimaging

Antibodies

Antibodies used

Antibodies used for flow cytometry are as follows: anti-mouse CD3-eFluor 450 (17A2) (Cat# 48-0032-82, 1:500), anti-mouse CD19-eFluor 450 (1D3) (Cat# 48-0193-82, 1:500), anti-mouse NKp46-PE (29A1.4) (Cat# 12-3351-82, 1:500), anti-mouse KLRG1-APC (2F1) (Cat# 17-5893-82, 1:500), anti-mouse CD127-PerCP-eFluor 710 (SB/199) (Cat# 46-1273-82, 1:500), anti-mouse IL-33R-PE (RMST2-2) (Cat# 12-9333-82, 1:500), anti-mouse NK1.1-APC (PK136) (Cat# 17-5941-82, 1:500), anti-mouse PD1-PE/Cyanine7 (J43) (Cat# 25-9985-82, 1:500), anti-mouse lineage cocktail-eFluor 450 (17A2; RB6-8C5; RA3-6B2; Ter-119; M1/70) (Cat# 88-7772-72, 1:500) and anti-mouse RORγt-APC (AFKJS-9) (Cat# 17-6988-82, 1:300) were purchased from Invitrogen; anti-mouse CD49a-PE/Cyanine7 (HMa1) (Cat# 142607, 1:500), anti-mouse α4β7-APC (DATK32) (Cat# 120607, 1:500), anti-mouse c-Kit-PE (2BB) (Cat# 105807, 1:500), anti-mouse IFN-γ-APC/Cyanine7 (XMG1.2) (Cat# 505849,1:500), anti-mouse Sca-1-FITC (W18174A, 1:500) (Cat# 160907) and anti-mouse CD45.2-FITC (30-F11) (Cat# 103107, 1:500) were from Biolegend.
Antibodies used for immunofluorescence are as follows: anti-mouse IL-7R (G-11) (Cat# sc-514445, 1:500) and anti-mouse T-bet (4B10) (Cat# sc-21749, 1:500) were from SantaCruz; anti-mouse CD3 (E4T1B) (Cat# 4443, 1:1000) was purchased from CST.
Secondary antibody conjugated to HRP: Anti-rabbit/Mouse (Cat# 10013001010)
Antibodies used for ChIP-qPCR are as follows: anti-mouse TET1 (5D6) (Cat# 61941) and Rat IgG1 Isotype control (TNP6A7) (Cat# BP0290)

Validation

All the antibodies are commercially available and validated by manufacturers for the indicated purpose.
anti-mouse CD3-eFluor 450 (17A2) (Cat# 48-0032-82, 1:500) https://www.thermofisher.cn/cn/zh/antibody/product/CD3-Antibody-clone-17A2-Monoclonal/48-0032-82,
anti-mouse CD19-eFluor 450 (1D3) (Cat# 48-0193-82, 1:500) https://www.thermofisher.cn/cn/zh/antibody/product/CD19-Antibody-clone-eBio1D3-1D3-Monoclonal/48-0193-82,
anti-mouse NKp46-PE (29A1.4) (Cat# 12-3351-82, 1:500) https://www.thermofisher.cn/cn/zh/antibody/product/CD335-NKp46-Antibody-clone-29A1-4-Monoclonal/12-3351-82,
anti-mouse KLRG1-APC (2F1) (Cat# 17-5893-82, 1:500) https://www.thermofisher.cn/cn/zh/antibody/product/KLRG1-Antibody-clone-2F1-Monoclonal/17-5893-82,
anti-mouse CD127-PerCP-eFluor 710 (SB/199) (Cat# 46-1273-82, 1:500) https://www.thermofisher.cn/cn/zh/antibody/product/CD127-Antibody-clone-eBioSB-199-SB-199-Monoclonal/46-1273-82,
anti-mouse IL-33R-PE (RMST2-2) (Cat# 12-9333-82, 1:500) https://www.thermofisher.cn/cn/zh/antibody/product/IL-33R-ST2-Antibody-clone-RMST2-33-Monoclonal/12-9333-82,
anti-mouse NK1.1-APC (PK136) (Cat# 17-5941-82, 1:500) https://www.thermofisher.cn/cn/zh/antibody/product/NK1-1-Antibody-clone-PK136-Monoclonal/17-5941-82,
anti-mouse PD1-PE/Cyanine7 (J43) (Cat# 25-9985-82, 1:500) https://www.thermofisher.cn/cn/zh/antibody/product/CD279-PD-1-Antibody-clone-J43-Monoclonal/25-9985-82,
anti-mouse lineage cocktail-eFluor 450 (17A2; RB6-8C5; RA3-6B2; Ter-119; M1/70) (Cat# 88-7772-72, 1:500) https://www.thermofisher.cn/cn/zh/antibody/product/Mouse-Hematopoietic-Lineage-Antibody-Cocktail/88-7772-72,
anti-mouse RORγt-APC (AFKJS-9) (Cat# 17-6988-82, 1:300) https://www.thermofisher.cn/cn/zh/antibody/product/ROR-gamma-t-Antibody-clone-AFKJS-9-Monoclonal/17-6988-82,
anti-mouse CD49a-PE/Cyanine7 (HMa1) (Cat# 142607, 1:500) https://www.biolegend.com/en-us/products/pe-cyanine7-anti-mouse-cd49a-antibody-16063,
anti-mouse α4β7-APC (DATK32) (Cat# 120607, 1:500) https://www.biolegend.com/en-us/products/apc-anti-mouse-lpam-1-integrin-alpha4beta7-antibody-6902,
anti-mouse c-Kit-PE (2BB) (Cat# 105807, 1:500) https://www.biolegend.com/en-us/products/pe-anti-mouse-cd117-c-kit-antibody-75,
anti-mouse IFN-γ-APC/Cyanine7 (XMG1.2, 1:500) (Cat# 505849) https://www.biolegend.com/en-us/products/apc-cyanine7-anti-mouse-ifn-gamma-antibody-13155,
anti-mouse Sca-1-FITC (W18174A) (Cat# 160907, 1:500) https://www.biolegend.com/en-us/products/fitc-anti-mouse-ly-6a-e-sca-1-antibody-21659,
anti-mouse CD45.2-FITC (30-F11) (Cat# 103107, 1:500) https://www.biolegend.com/en-us/products/fitc-anti-mouse-cd45-antibody-99,
anti-mouse IL-7R (G-11) (Cat# sc-514445, 1:500) https://www.scbt.com/zh/p/il-7r-antibody-g-11,
anti-mouse T-bet (4B10) (Cat# sc-21749, 1:500) https://www.scbt.com/zh/p/t-bet-antibody-4b10
anti-mouse CD3 (E4T1B) (Cat# 4443, 1:1000) https://www.cellsignal.cn/products/primary-antibodies/cd3e-cd3-12-rat-mab/4443
secondary antibody conjugated to HRP: Anti-rabbit/Mouse (Cat# 10013001010) https://u5mpootmgx.jiandaoyun.com/dash/611cc8d79bbb8a0008295ad3
anti-mouse TET1 (5D6) (Cat# 61941) https://www.activemotif.com.cn/catalog/details/61741/tet1-antibody-mab-clone-5d6,
rat IgG1 Isotype control (TNP6A7) https://bioxcell.com/invivoplus-rat-igg1-isotype-control-anti-trinitrophenol-bp0290.

Eukaryotic cell lines

Policy information about cell lines and Sex and Gender in Research

Cell line source(s)	OP9-DL1 cell line was a gift from Fan lab (Institute of Biophysics, Chinese Academy of Sciences).
Authentication	None of the cell lines were authenicated.
Mycoplasma contamination	OP9-DL1 cell lines was tested negative for mycoplasma contamination
Commonly misidentified lines (See ICLAC register)	None used

Animals and other research organisms

Policy information about studies involving animals; ARRIVE guidelines recommended for reporting animal research, and Sex and Gender in Research

Laboratory animals	WT C57BL/6 mice (body weight 15~20g, 2-8weeks old) were purchased from Beijing Vital River Laboratory Animal Technology Co., Ltd, China. Tet1flox/+ mice (C57BL/6 background) were from Shanghai Model Organisms Center, Inc. and crossed with Zbtb16-Cre mice(C57BL/6 background). Tet1flox/+;Zbtb16-Cre were crossed with Tet1flox/+ mice to obtain Tet1flox/flox;Zbtb16-Cre mice. Tet1flox/flox;Id2-Cre mice were generated with a similar strategy. Mice were maintained under specific pathogen-free conditions. Germ-free C57BL/6J mice (3-6 weeks old) were purchased from Department of Laboratory Animal Science, Peking University Health Science Center. Gnotobiotic C57BL/6J mice were maintained in germ-free isocages and fed with sterile food and water in the germ-free animal facility of Peking University. NOD.Cg-Prkdcscid IL2rgtm1Wjl/SzJ (NSG) mice (BALB/c background) were purchased from Biocytogen Pharmaceuticals Co., Ltd, China. Rorc-GFP mice (a gift from Guo XH, Tsinghua University)(C57BL/6 background) were maintained in specific-pathogen-free conditions. Animals were housed with 12/12h dark/light cycles, 23±2°C and 40% humidity conditions. Experiments were performed in accordance with protocols approval by the Institutional Committee of Institute of Microbiology, Chinese Academy of Sciences.
Wild animals	No wild animals were used in this study.
Reporting on sex	This study did not involve sex-based analysis. Both female and male mice were used in our experiments.
Field-collected samples	This study did not involve sample collected from the field.
Ethics oversight	All mouse procedure were approved by the Institutional Ethics Committee of Institute of Microbiology, Chinese Academy of Sciences. The study is compliant with all relevant ethical regulations regarding animal research.

Note that full information on the approval of the study protocol must also be provided in the manuscript.

Plants

Seed stocks	N/A
Novel plant genotypes	N/A
Authentication	N/A

Flow Cytometry

Plots

Confirm that:

- [x] The axis labels state the marker and fluorochrome used (e.g. CD4-FITC).
- [x] The axis scales are clearly visible. Include numbers along axes only for bottom left plot of group (a 'group' is an analysis of identical markers).
- [x] All plots are contour plots with outliers or pseudocolor plots.
- [x] A numerical value for number of cells or percentage (with statistics) is provided.

Methodology

Sample preparation

Intestines tissues from mice were cut open longitudinally and Peyer's patches were removed. Next, intestines were cut into small pieces for removing epithelial layers by incubation two times in 5 mM EDTA Ca2+ and Mg2+ free Hank's medium for 10 min each at 37°C, and the supernatants were collected for intraepithelial lymphocyte (IEL) analysis. Intestines tissues were then collected and cut into smaller pieces (1-2 mm3), followed by digesting for 60 min at 37°C with Collagenase II and III (1 mg/mL; Worthington), DNase I (200 mg/mL; Roche) on a rocking platform. The supernatants of digestive fluid were passed through a 100-μm cell strainer for removing undigested tissues pieces. The filtered fluid was collected in 50 mL tube and centrifuged at 2000 rpm for 5 min. The centrifuged cells were washed and resuspended with 1 mL FACS buffer (0.5% FBS of 1×PBS) for the following staining of antibodies.

Cell suspensions of liver or mesenteric lymph nodes (MLNs) were obtained by passing the tissues through a 100-μm cell strainer. Lung tissue was pre-digested with Collagenase II and III (1 mg/mL; Worthington), DNase I (200 mg/mL; Roche) and passed through a 100-μm cell strainer for obtaining a single cell suspension. Mouse bone marrow was collected from femurs by flushing with 1 mL FACS buffer, followed by passing the tissues through a 100-μm cell strainer for collecting cell suspensions. Cell suspensions were used for antibodies staining.

Cell surface markers (i.e. CD3, CD19, CD45.2, CD127, NK1.1, NKp46, KLRG1, ST2, CD49a, c-Kit, α4β7, PD1) were stained on ice for 60 min. For intracellular cytokine detection, cells were cultured in completed RPMI1640 media supplemented with ionomycin (500 ng/mL), PMA (50 ng/mL), and Brefeldin A (10 μg/mL) at 37°C for 4 h. Next, cells were collected for surface marker staining, and then fixed and permeablized by Intracellular Fixation & Permeablization buffer set (eBioscience), followed by intracellular antigen (i.e. IFN-γ and RORγt) staining.

Instrument

BD FACSAriaIII was used for purification and analysis

Software

FlowJO V10 was used for data analysis

Cell population abundance

Purity of isolated ILC cells was over 95% for each assay that was determined by post sorting analysis of flow cytometry.

Gating strategy

For flow cytometric analysis, ILC1s (ILC1=Lin-CD45+CD127+NK1.1+NKp46+, Lin=CD3, CD19, CD11b, Gr1, Ter119, CD45R), ILC2s (ILC2 = Lin-CD45+CD127+KLRG1+, Lin = CD3, CD19, CD11b, Gr1, Ter119, CD45R, NK1.1), ILC3s (ILC3=Lin-CD45+CD127 +RoRγt-GFP+, Lin= CD3, CD19, CD11b, Gr1, Ter119, CD45R, NK1.1), ILC1 precursors (ILC1Ps) (ILC1Ps = Lin-CD45+CD127 +NK1.1+NKp46+CD49a+, Lin CD3, CD19, CD11b, Gr1, Ter119, CD45R) and ILC2 precursors (ILC2Ps) (ILC2Ps=Lin-CD45+ST2 +KLRG1-, Lin=CD3, CD19, CD11b, Gr1, Ter119, CD45R). Zbtb16-GFPcre mice were used for isolation of ILCPs (ILCPs=Lin-c-Kit +CD127+α4β7+PLZF-GFP+, Lin=CD3, CD19, CD11b, Gr1, Ter119, CD45R) . Rorc-GFP mice were used for isolation of ILC3 precursors (ILC3Ps) (ILC3Ps=Lin-CD45+CD127+α4β7intRoRγt-GFP+, Lin = CD3, CD19, CD11b, Gr1, Ter119, CD45R). The following gating strategy was applied to eliminate non-specifically stained cells. First , lymphocyte gate was applied on the SSC-A / FSC-A window. Subsequently, sequential singlet/doublet discrimination was applied via SSC-W/SSC-H and FSC-W/ FSC-H. The cells populations were gated as above panel.

☒ Tick this box to confirm that a figure exemplifying the gating strategy is provided in the Supplementary Information.
